# Polymerase I as a Target for Treating Neurodegenerative Disorders

**DOI:** 10.3390/biomedicines12051092

**Published:** 2024-05-15

**Authors:** Mark S. LeDoux

**Affiliations:** 1 Department of Psychology and College of Health Sciences, University of Memphis, Memphis, TN 38152, USA; msledoux@memphis.edu or mledoux@veracityneuroscience.com; 2 Veracity Neuroscience LLC, Memphis, TN 38157, USA

**Keywords:** polymerase I, DNA, neurodegeneration, neuroregression, nucleolus, upstream binding transcription factor (UBTF)

## Abstract

Polymerase I (Pol I) is at the epicenter of ribosomal RNA (rRNA) synthesis. Pol I is a target for the treatment of cancer. Given the many cellular commonalities between cancer and neurodegeneration (i.e., different faces of the same coin), it seems rational to consider targeting Pol I or, more generally, rRNA synthesis for the treatment of disorders associated with the death of terminally differentiated neurons. Principally, ribosomes synthesize proteins, and, accordingly, Pol I can be considered the starting point for protein synthesis. Given that cellular accumulation of abnormal proteins such as α-synuclein and tau is an essential feature of neurodegenerative disorders such as Parkinson disease and fronto-temporal dementia, reduction of protein production is now considered a viable target for treatment of these and closely related neurodegenerative disorders. Abnormalities in polymerase I activity and rRNA production may also be associated with nuclear and nucleolar stress, DNA damage, and childhood-onset neuronal death, as is the case for the UBTF E210K neuroregression syndrome. Moreover, restraining the activity of Pol I may be a viable strategy to slow aging. Before starting down the road of Pol I inhibition for treating non-cancerous disorders of the nervous system, many questions must be answered. First, how much Pol I inhibition can neurons tolerate, and for how long? Should inhibition of Pol I be continuous or pulsed? Will cells compensate for Pol I inhibition by upregulating the number of active rDNAs? At present, we have no effective and safe disease modulatory treatments for Alzheimer disease, α-synucleinopathies, or tauopathies, and novel therapeutic targets and approaches must be explored.

## 1. Introduction

**Figure 1:**
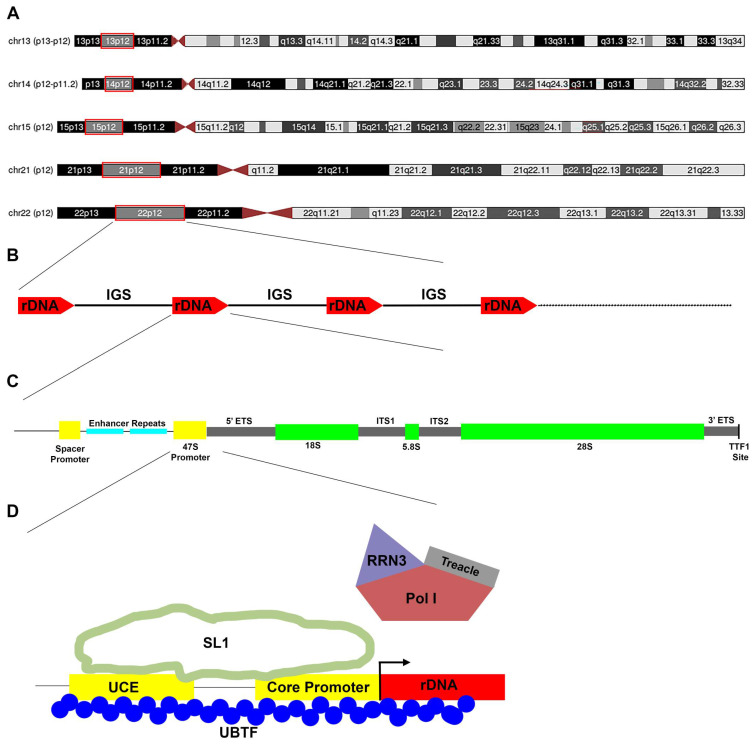
The five acrocentric chromosomes (**A**) that harbor tandem arrays of rDNA genes (**B**). The chromosomal localizations of the tandem repeats (RNA, ribosomal 45S clusters 1–5; RNR1, RNR2, RNR3, RNR4, and RNR5) as reported by the Human Gene Nomenclature Committee (genenames.org) are shown with red borders. The rDNA genes are organized from telomere to centromere. The total number of rDNA genes among these 10 acrocentric chromosomes and the total number of genes per individual chromosome vary among human populations, individuals, and tissues. In general, human rDNA genes are approximately 13 kb in length, and the Intergenic Spacers (IGS) are approximately 30 kb in length. The promoter region for each rDNA gene includes a spacer promoter, enhancer repeats, and 47S promoter (**C**). The promoter is followed from 5′ to 3′ by the 5′ external transcribed spacer (ETS), 18S rDNA, internal transcribed spacer 1 (ITS1), 5.8S rDNA, internal transcribed spacer 2 (ITS2), 28S rDNA, 3′-external transcribed spacer (3′ ETS), and transcription termination factor 1 (TTF1) binding site (**C**). The 47S promoter includes a core promoter and upstream control element (UCE) binding (**D**). Transcription requires a PIC consisting of UBTF and SL1. UBTF binds to the DNA and SL1 complex. The RRN3 association with Pol I facilitates interaction with the PIC. Treacle interacts with both Pol I and UBTF during the process of rDNA transcription.

Polymerase I (Pol I) is the rate-limiting enzyme in the production of ribosomes [1,2]. Ribosomes are intricate ribonucleoprotein complexes that translate mRNA into protein [3]. The production of ribosomes utilizes a high percentage of intracellular energy and resources. In non-neuronal eukaryotic cells, well over 50% of active transcription is devoted to rRNA [4,5]. In the nucleolus, Pol I transcribes a 47S rRNA precursor (Figure 1). Processing of this precursor into 28S, 18S, and 5.8S rRNA and pre-assembly of ribosomes also occur in the nucleolus [6,7]. Nucleolar assembly of ribosomes requires movement of ribosomal proteins, ribosomal biogenesis proteins, 5S rRNA, and snoRNAs into the nucleolus. Pre-assembled ribosomes are exported out of the nucleolus into the nucleus for further maturation and then into the cytoplasm for final maturation. Abnormalities in ribosomes, the rate of ribosomal biogenesis, and the quality and quantity of protein production play a role in numerous monogenic disorders, cancers, and neurodegeneration [8,9]. As such, upstream control of the rate-limiting enzyme Pol I is an attractive therapeutic target for a number of medical disorders.

There is significant pathobiological overlap among neurodegeneration, aging, and childhood-onset neuroregression. The nucleolus has been linked to premature and normal aging, and neuroregression [10]. Nucleolar abnormalities have also been identified in several neurodegenerative disorders. The nucleolus and Pol I may be viable targets to reduce the abnormal protein deposition characteristic of neurodegenerative disorders such as Parkinson and Alzheimer disease [11]. Manipulation of presumably normal nucleolar function and Pol I activity could be used as an upstream target to reduce protein production in physiologically stressed neurons. In addition, modest reductions in Pol I activity could potentially reduce nucleolar stress in disorders linked to aberrant production of pre-rRNA. 

## 2. The Nucleolus and Ribosomal DNA (rDNA)

The nucleolus is plurifunctional and harbors one to several hundred active rDNA copies, >100 small nucleolar RNAs required for rRNA processing, and >350 proteins [12]. Many of the proteins in the nucleolus do not end up in mature ribosomes. Numerous nucleolar proteins are involved in DNA repair, cell-cycle control, and signal recognition. The telomerase complex is assembled in the nucleolus, and telomere and rDNA silencing share factors important to genome stability [13,14]. For example, the action of two important cell-cycle regulators, p53 and MDM2, is regulated by sequestration in the nucleolus. Induction of rRNA synthesis and other nucleolar activities play a role in normal neural processes such as neurite outgrowth and memory consolidation during spatial training [15]. 

Nucleolar stress, which involves aberrant rRNA expression, has been associated with neurodegenerative disorders including Alzheimer disease and Parkinson disease [16,17] and, more recently, neuroregression [18]. Werner syndrome, characterized by premature aging and early death, is due to recessive LOF mutations in *WRN,* which encodes a DNA helicase localized in the nucleolus [19,20]. Rett syndrome, an X-linked neuroregression syndrome due to *MECP2* mutations, may also be due, in part, to nucleolar dysfunction [21]. MECP2 is a chromatin-associated protein that binds to methylated CpGs, including those associated with rDNA [22]. In preweanling mice, loss of MECP2 results in significantly smaller nucleoli and increased numbers of nucleoli in primary cortical neurons [23]. Overexpression of MECP2 in human 5Y cells results in larger nucleoli compared to the parental cell line [23]. Accordingly, longitudinal study of rRNA expression and nucleolar structure in mouse models may generate novel avenues for exploration of other neurodegenerative disorders, neuroregression syndromes, and aging. 

The nucleolus forms around active chromosomal rDNA arrays. Nucleolus number and size are dynamic processes closely tied to cell cycle regulation, rRNA production, and environmental cues. Clusters of rDNA arrays (nucleolus organizer regions, NORs) are present on human acrocentric chromosomes 13, 14, 15, 21, and 22 (Figure 1A). The haploid human and mouse genomes contain around 200 rRNA genes (rDNA) arranged in direct repeats at the NORs (Figure 1B). The NORs are subject to highly elevated levels of recombination and have been implicated in a number of genetic diseases, including Robertsonian and other chromosomal translocations [24]. Each cluster consists of multiple 45S rDNA repeat units that vary in number among individuals and chromosomes. The rDNA loci are arranged in telomere-to-centromere orientation (Figure 1B). Epigenetic control of rDNA involves a multitude of factors, including CpG methylation of rDNA, UBTF binding, and chromatin modifications [25,26]. Some NORs are permanently silenced by CpG methylation (meCpG). Active NORs correspond to visibly decondensed AgNOR loci (stained by silver) secondary constrictions in metaphase spreads, while meCpG-silenced rDNA corresponds to condensed NORs [27]. Tissue-specific methylation of rDNA may increase with aging and correlate with a decline in rRNA expression [28]. 

Acrocentric chromosomes are characterized by a non-central centromere (Figure 1A). Each of these chromosomes harbors many copies of rDNA, presumably to support high levels of rRNA and ribosome production, particularly during cellular proliferation and periods of increased physiological demand. Using 2546 human genomes from the 1000 Genomes Project, it has been estimated that the human rDNA copy number ranges from 61 to 1590 per diploid human genome (range: 61–1590, mean: 315) [29]. Other types of rDNA variation have been explored but incompletely characterized in the human brain: 5S rDNA position relative to 28S rDNA (S-type or L-type), single nucleotide coding and non-coding variants, retrotransposons, and structural variants (i.e., inversions) [30]. Moreover, particularly in the context of neurological disease and post-mitotic neurons in situ, little is known about rRNA transcription, ribosome production, ribosome stability, mosaicism, and ribosome turnover. All of these variables could impact the rate and fidelity of ribosome biogenesis and the response to inhibition of Pol I. 

## 3. Structure and Function of Pol I

A total of 13 genes encode proteins required for the formation of the 600 kDa Pol I complex (Table 1). The active center of Pol I is composed of RPA1 and RPA2. Five Pol I subunits (RPABC1, RPABC2, RPABC3, RPABC4, and RPABC5) are common to all three polymerases (Pol I, Pol II, and Pol III). Pol II transcribes mRNAs, small nuclear RNAs (snRNAs), and microRNAs. Pol III transcribes 5S rRNA and tRNA. RPA1 harbors numerous functional domains (from N-terminal to C-terminal: clamp, active center, pore, funnel, cleft, foot, jaw, expander, jaw, cleft, and clamp) [1,31,32]. RPA2 is a smaller protein with protrusion, lobe, fork, hybrid binding, wall, and clamp domains. DNA is loaded into the cleft, localized between the clamp and protrusion domains. RPA49 and RPA34 form a heterodimer that contributes to initiation and elongation. Cryo-electron microscopy (Cryo-EM) structural studies suggest that RPA43 functions in termination, open complex formation, elongation, and termination [33]. 

Pol I exhibits a high initiation rate and elongation speed [34]. In yeast, the overall initiation rate, not the number of active rDNA genes, determines rRNA transcription rates [34]. Over 100 Pol I molecules may be simultaneously active on a single rDNA gene. Pol I transcription is regulated by post-translational modification of components of the pre-initiation complex and changes in the number of active rDNA genes via epigenetic mechanisms. 

Silent rDNA exists in a closed heterochromatin state, whereas active rDNA exists in the euchromatin state. Epigenetic states are controlled by cycle-cells, environmental factors, and total rDNA copy number [35,36]. The epigenetics of rDNA regulation in terminally differentiated neurons are difficult to study and poorly understood. Moreover, the study of rDNA in the brains of animal models such as mice may be a poor surrogate for the aged adult human brain. 

## 4. Pol I Interactions

The synthesis of rRNA (Figure 1C) by Pol I is critically dependent on transcription and termination factors (Table 2), particularly the Upstream Binding Transcription Factor (UBTF). UBTF binds to the Pol I Upstream Control Element (UCE, −200 to −107) and core element (−45 to +20) (Figure 1D). UBTF is a multi-HMGB (High Mobility Group B)-box architectural DNA binding protein essential for rRNA transcription by Pol I and for ribosome biogenesis in the nucleolus [26,37]. UBTF exists as two isoforms, UBTF1 and UBTF2. The ratio of UBTF1 to UTBF2 is correlated with the fraction of active rDNA. 

UBTF contains a dimerization domain and six tandem HMGB boxes, the first three of which bind in the minor DNA groove and induce bending [38,39,40]. UBTF replaces histone chromatin across active rRNA genes, inducing a 16 kbp long Nucleosome-Free Region (NFR) [27]. It is also found genome-wide in GC rich NFRs adjacent to nucleosomes containing the H2A.Z histone variant. These NFRs also lie immediately upstream of RNA polymerase 2 (Pol II)-transcribed mRNA genes implicated in chromatin formation and cell cycle progression that have been suggested to be regulated by UBTF [41]. 

UBTF1 and UBTF2 are present across rDNA and at NFRs genome-wide as hetero- and homo-dimers, but only UBTF1 can cooperate with the TBP-complex SL1 to form the preinitiation complex. Thus, UBTF1 is essential for rDNA transcription, but UBTF1 and UBTF2 are equivalent for NFR formation and potentially for Pol II regulation. Given its critical role in rDNA transcription and ability to stabilize NFRs, knock-down of UBTF has been shown to cause genome instability [41]. Defects in ribosome biogenesis due to knockdown of UBTF lead to the stabilization of p53 and nucleolar stress and, consequently, cell cycle arrest and apoptosis [41,42,43,44,45,46]. Loss or mutation of UBTF could affect the formation of both rDNA-specific and genome-wide NFRs, possibly leaving the underlying DNA poorly protected and explaining the enhanced damage that has been observed. Extrapolating from yeast studies of the ortholog Hmo1, it is likely that UBTF-dependent NFRs are setup during genome replication [47]. Loss of UBTF induces enhanced H2A.Z acetylation, a marker of H2A.Z histone turnover, adjacent to associated NFRs, indicating increased chromatin instability at these sites [26]. Thus, mutations in UBTF could not only cause genome instability by inducing nucleolar stress but also by inducing NFR instability. 

Pol I recruitment to rDNA requires a pre-initiation complex (PIC) consisting of UBTF and SL1. UBTF binds to the SL1 complex (Table 2), composed of the TATA-binding protein (TBP) and four TBP-associated factors (TAF1A, TAF1B, TAF1C, and TAF1D). RRN3 associates with Pol I and enables interaction with the PIC. Once Pol I clears the promoter, the PIC remains bound and ready to recruit another Pol I molecule. Transcription termination factor 1 (TTF-1) binds to a consensus terminator element downstream of the 3′ end of pre-rRNA and mediates the termination of pre-rRNA synthesis. In theory, drugs that target interactions of Pol 1 with rDNA or its transcription factors could alter the production of rRNA. 

## 5. Pol I Inhibitors

The molecular mechanisms by which putative “Pol I inhibitors” operate remain poorly understood. Possible mechanisms include inhibition of elongation, prevention of promoter escape during initiation, and activation of a DNA damage response [48,49,50]. Drug classes include DNA intercalators, G4-stabilizers, TOP2 (Topoisomerase 2) inhibitors, DNA crosslinkers, and TOP1 (Topoisomerase 1) inhibitors [2]. Numerous Pol I inhibitors have been reported in the literature, but only a few have made it to clinical trials or regulatory approval [2].

Actinomycin D {ActD; 2-Amino- 4,6-dimethyl- 3-oxo- 3H-phenoxazine- 1,9-dicarboxylic acid bis- [(5,12-diisopropyl- 9,13,16-trimethyl- 4,7,11,14,17-pentaoxo- hexadecahydro- 10-oxa- 3a,6,13,16-tetraaza- cyclopentacyclohexadecen- 8-yl)- amide]; C_62_H_86_N_12_O_16_} inhibits Pol I and, at higher dosages, Pol II transcript elongation [51]. ActD reduces the production of rRNA [52]. ActD is used to treat a variety of malignant tumors, including Wilms tumor, Ewing sarcoma, testicular cancer, and trophoblastic neoplasms. Typically, ActD is administered intravenously every 2–3 weeks. ActD shows poor CNS penetration. 

BMH-21 {N-(2-(Dimethylamino)ethyl)-12-oxo-12H-benzo[g]pyrido [2,1-b]quinazoline-4-carboxamide; C₂₁H₂₀N₄O₂} is a planar heterocyclic small molecule DNA intercalator that binds strongly to GC-rich DNA sequences, ultimately inhibiting Pol I, blocking transcription, and disrupting nucleolar structure [53]. BMH-21 penetrates the CNS and has been used in murine preclinical studies of spinal cord injury [54], but is not being used in clinical studies at present due to deleterious off-target effects.

CX-5461 {2-(4-Methyl-1,4-diazepan-1-yl)-N-((5-methylpyrazin-2-yl)methyl)-5-oxo-5H-benzo [4,5]thiazolo [3,2-a][1,8]naphthyridine-6-carboxamide; C_27_H_27_N_7_O_2_S} is an orally bioavailable small molecule that selectively inhibits Pol I-driven transcription relative to Pol II-driven transcription (~200-fold in human cell lines) [55,56,57]. CX5461 was initially claimed to inhibit Pol I via disruption of the SL1-rDNA complex, but the Moss lab has now shown that it actually blocks initiation [50]. CX5461 shows limited CNS penetration. CX5461 is currently in Phase I/II clinical testing (NCT02719977) for solid malignancies. 

Orally-bioavailable, improved 2nd-generation Pol I inhibitors that are orally-bioavailable are in early phase clinical trials. PMR-116 is one such example. PMR-116 induces phosphorylation and accumulation of p53 and does not activate CHK2 [58]. PMR-116 is currently in a Phase I dose escalation trial in patients with solid tumors (ACTRN12620001146987). PMR-116 has shown efficacy in MYC-driven cancer models. More specifically, these small molecules have been tested in preclinical models of metastatic breast cancer [59]. At this time, the chemical formula for PMR-116 is not available in the public domain.

## 6. Human Mutations with Direct Effects on the Pol I Enzymatic Complex

**Figure 2:**
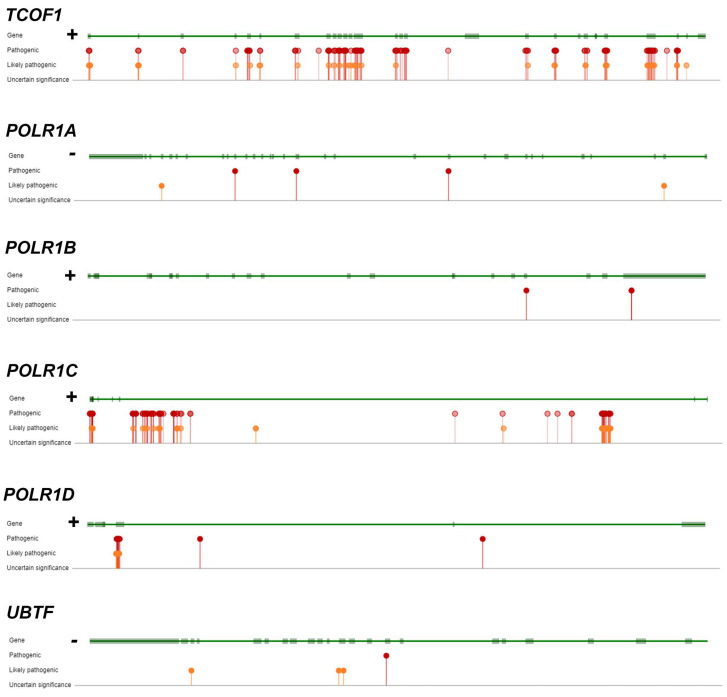
The genomic localizations of ClinVar pathogenic and likely pathogenic single nucleotide variants in *TCOF1*, *POLR1A*, *POLR1B*, *POLR1C*, *POLR1D*, and *UBTF*. Gene orientation (+ or -) is included to the left of each gene’s intron/exon structure.

Most cases of Treacher Collins Syndrome (TCS) are caused by autosomal dominant mutations (Table 2), typically de novo, of *TCOF1,* which encodes treacle (TCS1) [60]. Treacle recruits Pol I and UBTF to the rDNA promoter [61] (Figure 1). A total of 196 pathogenic or likely pathogenic *TCOF1* variants are reported in ClinVar (https://www.ncbi.nlm.nih.gov/clinvar/, accessed on 1 May 2024). The vast majority are short variants (<50 bp). Variants are distributed throughout *TCOF1* (Figure 2). Variation type includes deletions (*n* = 89), duplications (*n* = 35), indels (*n* = 5), insertions (*n* = 34), and single nucleotide (*n* = 50). The majority of variants lead to frameshifts.

TCS can also be caused by mutations in *POLR1B* (TCS4), *POLR1C* (TCS3), and *POLR1D* (TCS2) (Table 1). Reported mutations in TCS3 are autosomal recessive. TCS2 may be dominant or recessive. Since *POLR1C* and *POLR1D* are shared with Pol III, the effects of mutations in *POLR1C* and *POLR1D* cannot be attributed to Pol I dysfunction in isolation (Table 1). 

TCS4 is dominant. A total of 15 pathogenic or likely pathogenic variants in *POLR1B* are reported in ClinVar. Two missense variants are recurrent: NM_019014.6(POLR1B):c.2046T>A (p.Ser682Arg) and NM_019014.6(*POLR1B*):c.3007C>A (p.Arg1003Ser) (Figure 2). 

TCS2 is most commonly autosomal dominant due to heterozygous nonsense or missense mutations [62]. Autosomal recessive TCS2 has also been described in two unrelated consanguineous families with the identical missense *POLR1D* variant. 

TCS is characterized by severe craniofacial structural abnormalities that arise in utero. Phenotypic features may include malformed auricles, malar and mandibular hypoplasia, conductive hearing impairment, down slanting palpebral fissures, the absence of the lower eyelashes, and, rarely, a cleft palate [63]. In general, patients with TCS1 show no intellectual disability. Some patients with TCS2 may show delays in motor and speech development. TCS penetrance may be incomplete, and subtle phenotypes have been reported. Many TCS patients require major reconstructive facial surgery. 

Autosomal dominant mutations in *POLR1A* cause Acrofacial Dysostosis, Cincinnati Type (AFDCIN) [64]. AFDCIN shares some features with TCS but, in general, is more severe, and affected individuals may also manifest short stature, microcephaly, bowed forearms, radial aplasia, heart defects, and neurological dysfunction (developmental delay, epilepsy, infantile spasms, and hypotonia) [65]. ClinVar reports 15 pathogenic and 3 likely pathogenic variants in *POLR1A* (Figure 2) including 11 deletions, 4 duplications and 3 single nucleotide variants leading to missense, frameshift, and nonsense molecular consequences. 

Ostensibly autosomal recessive hypomyelinating leukodystrophy (HLD) has been linked to autosomal recessive mutations in *POLR1A* (HLD27) and *POLR1C* (HLD11) (Table 1). Reported mutations in *POLR1A* resulted in homozygous variants (NM_015425.3, c.2801C-T, p.S934L; and NM_015425.3, c.1925C-A, p.T642N). HDL11 mutations in *POLR1C* may be homozygous or compound heterozygous [66]. HLD may be a misnomer since careful analysis of clinical descriptions indicates that the hypomyelination reported in patients with deleterious *POLR1A* variants is secondary to neuronal loss since affected individuals show clear magnetic resonance imaging (MRI) evidence of global neurodegeneration with reductions in both gray and white matter volumes. Patients show neurodevelopmental abnormalities with a later appearance of neuroregression [67,68]. Reported neurological findings include intellectual disability, ataxia, dystonia, oculomotor abnormalities, seizures, and spasticity. Similar findings have been reported in individuals with homozygous or compound heterozygous *POLR1C* mutations, which would impact both Pol I and Pol III [65,66]. Some patients with *POLR1C* mutations also have dental abnormalities. 

In aggregate, analysis of mutations that directly affect Pol I suggests that (1) Pol 1 inhibition should be avoided during early development, (2) some degree of Pol 1 inhibition may be tolerated after the early prenatal period of life, and (3) partial Pol I inhibition may be associated with few adverse neurological effects in adults. 

## 7. Human Mutations with Indirect Effects on Pol I

The UBTF E201K neuroregression syndrome is a recurrent de novo dominant mutation most commonly associated with neurodegeneration beginning at 2.5 years of age [69,70]. In some patients, neuroregression is superimposed on mild developmental delay. Other *UBTF* variants have been reported in patients with similar clinical syndromes (Figure 2) [71,72]. The term CONDBA (childhood-onset neurodegeneration with brain atrophy) has been used as a more general term for all pathogenic UBTF variants because MRI imaging in affected subjects shows progressive loss of brain volume [70]. Affected individuals become non-ambulatory and aphasic by their early teen years. Neurological features include ataxia, dystonia, intellectual impairment, dysarthria, and dysphagia. Seizures and Parkinsonism have been reported. In some cases [73,74]. Overall, there is substantial clinical and neuroimaging overlap among patients with *UBTF*, *POLR1A,* and *POLR1C* mutations. 

The TATA box-binding protein (TBP), which contributes to the SL1 complex, has been linked to spinocerebellar ataxia type 17 (SCA17). SCA17 is also known as Huntington Disease-Like 4 (HDL4). Most commonly, SCA17 is associated with CAG/CAA repeat expansions in *TBP* [75]. Some cases are digenic due to intermediate expansions in *TBP* coupled with mutations in *STUB1*. SCA17 typically presents during adult life with variably progressive combinations of cerebellar ataxia, cognitive decline, seizures, chorea, and dystonia. *TBP* is a general transcription factor required for Pol I, polymerase II (Pol II), and Polymerase III (Pol III). To my knowledge, the quantitative effects of TBP repeat expansions on Pol I activity and synthesis of rRNA are not known, and the acquisition of this data would be one starting point for targeted therapeutics. 

## 8. Targeting Neurodegeneration, Neuroregression, and Aging

Numerous neurodegenerative disorders are due, at least in part, to the accumulation of proteins in the central nervous system (CNS). Protein accumulation may predominate in certain cell types of the CNS, with little or no obvious cellular pathology in extra-neural tissues. Here, we will provide examples of CNS disorders associated with protein accumulation and/or nucleolar dysfunction. In theory, Pol I could be viewed as an upstream target to reduce the pathological accumulation of wild-type and mutant proteins and/or reduce nucleolar stress.

In Parkinson disease, α-synuclein encoded by *SNCA*, accumulates in neurons, typically with early involvement of monoaminergic neurons of the locus coeruleus and substantia nigra, pars compacta. Prior to the onset of motor dysfunction, the majority of patients destined to develop Parkinson disease manifest one or more non-motor features such as hyposmia, constipation, or REM sleep behavior disorder. Moreover, deleterious variants in *LRRK2* and *GBA* increase the risk of developing Parkinson disease. As such, there are both clinical and genetic markers that can be used to predict the risk of developing Parkinson disease and permit early intervention with disease-modulating therapeutics prior to penultimate clinical decline. Nucleolar dysfunction involving p53 and mTOR signaling may contribute to the pathobiology of Parkinson disease [76]. Another α-synucleinopathy, multiple system atrophy, is characterized by the deposition of α-synuclein in oligodendroglia. 

Tauopathies are a collection of neurodegenerative diseases with both specific and overlapping clinical features that are histopathologically characterized by abnormal accumulation and aggregation of tau within neurons, glia, or both. Tau, encoded by *MAPT*, is a microtubule associated protein. Alternative splicing of the *MAPT* transcript generates 6 CNS isoforms with different numbers of N-terminal inserts and either 3 or 4 repeats (3R or 4R) in the repeat domain. Tauopathies are often divided into primary and secondary forms. Primary forms include frontotemporal lobar degeneration, corticobasal degeneration, and progressive supranuclear palsy. Secondary forms include Alzheimer disease (AD) and chronic traumatic encephalopathy. At the microscopic pathological level, these disorders differ in the relative involvement of neurons, astrocytes, and oligodendroglia. At the molecular level, they differ in the relative accumulation of 3R and 4R tau. Tau is found in both the nucleus and cytoplasm. In the nucleolus, tau may be involved in silencing rDNA [77].

The spinocerebellar ataxias (SCAs) are a diverse group of inherited disorders mainly characterized by cerebellar atrophy and ataxia on clinical examination. To date, more than 50 autosomal dominant SCAs have been reported in the medical literature, and many of the more prevalent SCAs are trinucleotide repeat disorders. SCA1, SCA2, SCA3, SCA6, SCA7, SCA12, and SCA17 are due to CAG repeat expansions. Another movement/neurodegenerative disorder, Huntington disease (HD), is also caused by CAG repeat expansions. Expanded polyQ proteins from insoluble cellular aggregates [78]. In addition, some ataxia-associated polyQ proteins localize to the nucleolus and may impact nucleolar function [79].

AD pathology is characterized by the aggregation of amyloid beta (Aβ) proteins into extracellular plaques and tau into intraneuronal neurofibrillary tangles. AD pathology is commonly seen in postmortem brains of aged individuals without clinical evidence of overt cognitive impairment. Other pathologies, particularly limbic-predominant age-related TDP-43 encephalopathy (LATE) neuropathological change (LATE-NC), are commonly found in the brains of aged individuals and may contribute to minor deterioration in motor, sensory, and cognitive abilities commonly seen as part of the so-called normal aging process [80]. Repeat expansions of *C9orf72* are the most common known genetic cause of amyotrophic lateral sclerosis (ALS). In both C9orf72-associated and sporadic ALS, nucleolar stress appears to be upstream of pathological disease hallmarks, specifically TDP-43 mislocalization and antisense RNA foci [81].

UBTF-associated neuroregression is a potential target for treatment with Pol I inhibition. Mutant UBTF E210K shows gain of function with increased binding to the rDNA promoter and 5′-external transcribed spacer [69]. Patient UBTF E210K fibroblasts showed increased expression of pre-rRNA (>3X) and 18S rRNA (2X) [70]. Mutant fibroblasts tended to have fewer nucleoli per nucleus and increased numbers of 53BP1-foci [70]. The UBTF E210K fibroblasts showed increased apoptosis and abnormal progression to the G2 phase of the cell cycle [70]. *Ubtf*^−/−^ is early embryonic lethal in mice [82], and transgenic expression of human UBTF1 E210K in Drosophila neurons is also lethal [70].

Nucleolar stress due to excessive, unnecessary, or aberrant Pol I activity and ribosomal synthesis has also been associated with normal aging and premature aging syndromes such as progeria. Disturbed ribosomal biogenesis and enlarged nucleoli are seen in patients with Hutchinson-Gilford Progeria Syndrome and fibroblasts from aged humans [83]. With aging, the accumulation of somatic mutations in nuclear (nDNA) and mitochondrial DNA (mtDNA) leads to the production of mutant proteins of little or no functional value, leading to a deterioration of multiple cellular processes and the cellular burden of eliminating unwanted proteins. Similarly, the accumulation of mutations in rDNA would lead to the production of aberrant rRNA and ribosomal pathology. Intrinsic and extrinsic triggers of nucleolar stress also lead to a cascade of secondary effects, including damage to non-nucleolar nDNA. It should also be emphasized that ribosome biogenesis, both normal and aberrant, is energy demanding, which, for disorders such as Parkinson disease that have been linked to mitochondrial dysfunction, is particularly undesirable. In this context, it is important to note recent work showing that reducing the metabolic burden of rRNA synthesis promotes longevity in *C. elegans* [35]. 

Gene therapy, particularly antisense oligonucleotides, has been employed in clinical trials of neurodegenerative disorders. The complications of delivery strategies and blood barrier penetration have been limited. For instance, a trial of tominersen in HD, an antisense oligonucleotide delivered via intrathecal injection, was stopped due to a lack of efficacy [84]. Despite technological improvements, many critical issues remain, particularly in the context of long-term delivery, and these include antibody formation, inflammatory response, reduction of wild-type protein, penetration of deep brain tissues, and cost. Small molecule enzyme inhibitors that cross the blood-brain barrier, such as rasagiline (monoamine oxidase type B) for Parkinson disease and donepezil (acetylcholinesterase) for Alzheimer disease, have proven to be efficacious and well tolerated by patients for years to decades. The same could apply to Pol I inhibition. 

## 9. Unanswered Questions

The ideal Pol I inhibitor to treat aging and neurodegenerative/neuroregression disorders of the CNS should cross the blood-brain barrier, have no effect on Pol II or Pol III, and have no deleterious off target effects or toxicity. More specifically, is it possible to effectively target post-mitotic neurons when Pol I is critical for the survival of extra-neural tissues such as the rapidly dividing cells of the hematopoietic system? Preliminary studies can be done in cells, ideally cultured neurons or human-induced pluripotent stem cells (hiPSCs). Next level studies can use worms (*C. elegans*) and flies (*Drosophila*). Mice are the most practical mammalian system, but they live less than two years. Mice have often proved to be poor models for late-onset human neurodegenerative disorders and aging. Ultimately, human studies will be required for validation. As always, Phase I and II studies on humans must focus on safety. Efficacy studies (Phase III) will likely require prolonged interventions lasting at least one year. Shorter studies of disease-modifying therapeutics in Parkinson disease and Huntington disease have failed to reach predefined clinical endpoints [85,86].

Implementation of Pol I inhibition for the treatment of neurodegeneration, neuroregression, and aging will require answers to several key questions. First, would chronic or intermittent inhibition of Pol I lead to compensatory increases in active rDNA or trigger nucleolar stress? How will Pol I inhibition alter the kinetics of target (e.g., α-synuclein) and off-target protein production and clearance? What are the downstream effects of Pol I inhibition that may be largely independent of protein production? How would chronic, mild inhibition of Pol I affect other aspects of nucleolar function? How would the pharmacokinetics and pharmacodynamics of an orally-available Pol I inhibitor inform dosing strategies? Dosing strategies (twice daily, daily, weekly, monthly, or other) may also be dictated by a specific disease state. Effective and inexpensive disease-modifying drugs to slow the progression of neurodegeneration, neuroregression, and aging could transform healthcare, and partial Pol I inhibition is one pharmacological approach that warrants evaluation.

## Figures and Tables

**Table 1:** Polymerase I (Pol I).

Subunit Protein	Gene Symbol	Shared with Pol II	Shared with Pol III	OMIM Associated Disorders
RPA1	*POLR1A*	No	No	AD: Acrofacial dysostosis, Cincinnati type AR: Leukodystrophy, hypomyelinating, 27
RPA2	*POLR1B*	No	No	AD: Treacher Collins syndrome 4
RPAC1	*POLR1C*	No	Yes	AR: Leukodystrophy, hypomyelinating, 11 AR: Treacher Collins syndrome 3
RPAC2	*POLR1D*	No	Yes	AD/AR: Treacher Collins syndrome 2
RPA49	*POLR1E*	No	No	None
RPA43	*POLR1F*	No	No	None
RPA34	*POLR1G*	No	No	None
RPA12	*POLR1H*	No	No	None
RPABC1	*POLR2E*	Yes	Yes	None
RPABC2	*POLR2F*	Yes	Yes	None
RPABC3	*POLR2H*	Yes	Yes	None
RPABC4	*POLR2K*	Yes	Yes	None
RPABC5	*POLR2L*	Yes	Yes	None

AD, autosomal dominant. AR, autosomal recessive.

**Table 2:** Pol I Regulatory Factors.

Protein	Gene Symbol(s)	Function	OMIM Associated Disorders
UBTF	*UBTF*	recruitment of Pol I to rDNA, determining a specialized non-nucleosomal chromatin structure on active rDNA, cooperating with other components of the pre-initiation complex at the Pol I promoter	UBTF E210K neuroregression syndrome (AKA—neurodegeneration, childhood-onset, with brain atrophy [CONDA], hematological malignancies
SL1 complex (TBP, TAF1A, TAF1B, TAF1C, TAF1D)	*TBP*, *TAF1A*, *TAF1B*, *TAF1C*, *TAF1D*	essential component of the pre-initiation complex, interacts with UBTF and Pol I	*TBP* (AD: spinocerebellar ataxia 17) TAF1A—none TAF1B—none TAF1C—none TAF1D—none
RRN3	*RRN3*	mediates the interaction of Pol I with UBTF and SL1	None
TTF1	*TTF1*	terminates Pol I transcription	None
TCOF1 (Treacle protein)	*TCOF1*	Pol 1 rDNA promotor recognition and recruitment of UBTF	AD: Treacher Collins syndrome 1

AD, autosomal dominant.
